# A 35-Year Trend Analysis for Back Pain in Austria: The Role of Obesity

**DOI:** 10.1371/journal.pone.0107436

**Published:** 2014-09-10

**Authors:** Franziska Großschädl, Wolfgang Freidl, Éva Rásky, Nathalie Burkert, Johanna Muckenhuber, Willibald J. Stronegger

**Affiliations:** 1 Medical University of Graz, Institute of Social Medicine and Epidemiology, Graz, Austria; 2 University of Graz, Institute of Sociology, Graz, Austria; Dasman Diabetes Institute, Kuwait

## Abstract

**Background:**

The prevalence of back pain is constantly increasing and a public health problem of high priority. In Austria there is a lack of empirical evidence for the development of back pain and its related factors. The present study aims to investigate trends in the prevalence of back pain across different subpopulations (sex, age, obesity).

**Methods:**

A secondary data analysis based on five nationally representative cross-sectional health surveys (1973–2007) was carried out. Face-to-face interviews were conducted in private homes in Austria. Subjects aged 20 years and older were included in the study sample (n = 178,818). Obesity was defined as BMI≥30 kg/m^2^ and adjusted for self-report bias. Back pain was measured as the self-reported presence of the disorder.

**Results:**

The age-standardized prevalence of back pain was 32.9% in 2007; it was higher among women than men (p<0.001), higher in older than younger subjects (p<0.001) and higher in obese than non-obese individuals (p<0.001). During the investigation period the absolute change in the prevalence of back pain was +19.4%. Among all subpopulations the prevalence steadily increased. Obese men showed the highest increase of and the greatest risk for back pain.

**Conclusion:**

These results help to understand the development of back pain in Austria and can be used to plan controlled promotion programs. Further monitoring is recommended in order to control risk groups and plan target group-specific prevention strategies. In Austria particular emphasis should be on obese individuals. We recommend conducting prospective studies to confirm our results and investigate causal relationships.

## Introduction

Back pain represents an extremely common public health problem [1]–[3] and is especially widespread in Western countries [4]. A systematic review investigating the global prevalence of activity-limiting low back pain among adults estimated a point prevalence to range from 1.0% to 58.1% (mean 18.1%) and a 1-year prevalence from 0.8% to 82.5% (mean 38.1%). Back pain has a negative impact at the individual level, e.g. through strong pain and activity limitations [2], and at the social level, e.g. through absenteeism, the need for disability pension [5]–[7], a high utilization of health care resources, and other financial aspects [5], [8], [9]. Given the high total economic costs of back pain, even a small reduction in the incidence of back pain would have a sustained economic impact [10]. A whole range of environmental and personal factors must be considered when investigating back pain as a disorder. Studies have found e.g. age [7], [11], [12], sex [2], [7], [12] and BMI [13] to present a significant association with the risk of suffering from back pain.

An association between obesity and the presence of back pain has been reported [10], [14]–[17]. Compared to individuals with normal weight, subjects with obesity more often self-report a poorer health status [18]. It has been observed that the morbidity and mortality risk increase with increasing body mass index (BMI kg/m^2^) [19]. While the prevalence of obesity increased strongly worldwide over the last decades, there was also a clear parallel upward trend in the prevalence of different obesity-associated diseases and disorders, such as type 2 diabetes mellitus, cardiovascular diseases, malignant tumours or back pain [20]. A large Austrian population-based study showed a strong upward trend in the mean BMI and the prevalence of obesity among adult women and men. At present the age-standardized obesity prevalence is estimated to be 14.5% and seems to be rising among Austrian adults [21].

There is a lack of information regarding the development of back pain in Austria and its relation to obesity. Evidence-based confirmation is still lacking. A representation of existing long-term trends for back pain could demonstrate the extent of this problem in Austria and the investigation of subgroups would furthermore allow to identify the factors behind back pain-affected populations and detect special risk groups [22]. This would facilitate the planning of target group-specific preventive measures and reduce the number of people affected by back pain. In addition, the monitoring of secular back pain trends can be utilised to evaluate prevention strategies.

The purpose of this study was to demonstrate the changes in back pain trends among the Austrian adult population in the period 1973 to 2006–07. Long-term trends in the prevalence of back pain were to be presented for Austria as a whole and for different subpopulations (based on sex, age, and obesity). This study also aimed to identify possible risk groups by assessing the associations of back pain with collected variables.

## Methods

### Data source and sampling

Data were derived from representative cross-sectional health surveys carried out in Austria using comparable methodology. Since 1973, five nationwide health surveys have been conducted at irregular time intervals. Health data were collected through the Austrian Microcensus in 1973, 1983, 1991 and 1999. The last health survey – titled Austrian Health Interview Survey (AT-HIS 2006–07) – was conducted in 2006–07 instead of the former Microcensus on health as part of the European Health Interview Survey (E-HIS; http://www.euhsid.org), an important high-quality survey. The Microcensus and the AT-HIS are surveys conducted by the federal statistical office ‘Statistik Austria’. Statistics Austria (http://www.statistik.at/web_en/) is the owner of the data and makes them available. The Microcensus data are chargeable and the data for the ATHIS are free.

For the AT-HIS 2006–07 a random sample was drawn from the Austrian population register. For the sake of representation, the sample was stratified by the 32 administrative Austrian districts. For the four Microcensus surveys the sampling was made by a stratified selection of addresses by federal states. The selection framework for the Microcensus sampling was the housing census revised by the current housing statistics in Austria.

In all five surveys data were obtained through standardised face-to-face interviews by trained interviewers of ‘Statistik Austria’ questioning individuals aged 15 years and older in their private homes or long-term care facilities (such as nursing homes), using interviewer questionnaires. While a household sample was selected for the Microcensus surveys (this means that data from all household members were collected), a sample of the respective individuals was interviewed for the AT-HIS 2006–07. Another difference between Microcensus and AT-HIS is that in the AT-HIS, the participants were questioned by computer-assisted personal face-to-face interviews (CAPI), which allows direct data entry. To ensure that interviews were conducted in the same way, interviewers of all five surveys had to participate in trainings where they were instructed on how to conduct the interviews. In all five surveys participants had to give full information for the baseline survey portion. The raw data were screened for errors from ‘Statistik Austria’.

The participation rates were quite large for the Microcensus surveys, especially in 1973, and relatively low in the AT-HIS 2006/07. This is due to the fewer number of questions asked in the first surveys. The questionnaire applied in the AT-HIS was much more extensive in comparison with the questionnaires of the earlier surveys. However, each survey sample was weighted according to sex, age and region to ensure representativeness of the Austrian population distribution.

Data analysis for this study was limited to adults. Subjects aged 20 years and older were included since the AT-HIS survey rather concerned entire age groups (5 year intervals) than exact age levels. Therefore, the data of 64,052 subjects were excluded since they were younger than 20 years at the time of the survey. Furthermore, cases with missing data regarding gender and BMI were not included (n = 29,709). Cases with implausible BMI values (BMI≤10 kg/m^2^, BMI≥75 kg/m^2^) were also removed from the data base. This reduced the total sampling frame to 178,818 individuals. The proportion of individuals included in the analysis was 63% in total. 53.7% of the participants were female. The mean±SD age of the individuals was 47.7±17.5 years, which refers only to the first four surveys. The subjects included in this study were between 20 and 99 years old.

### Ethical approval

The consent procedure and the conductance of this study were approved by the Ethics Committee of the Medical University of Graz (EK-number: 23–172 ex 10/11). The study was carried out in compliance with the principles laid down in the Helsinki Declaration. No minors or children were included in the study sample. Data were collected anonymously. Verbal informed consent was obtained from all subjects, witnessed, and formally recorded for every survey.

### Variables and measurement

Demographic and socioeconomic characteristics, as well as health data were collected in each health survey. In every survey the presence of back pain was queried. When collecting the data in the Microcensus surveys (1973, 1983, 1991, 1999) participants were asked if they suffered from back pain at the time of the survey In the AT-HIS 2006–07 data for back pain were collected by asking the participants if they suffered from the disorder within the last 12 months. The surveys used different definitions for identifying back pain. In the last survey the 12-month prevalence of back pain was collected, while in the first four Microcensus surveys the point prevalence was measured. Despite the different collection methods in the Microcensus and the AT-HIS it was reported that back pain is often chronic [2] and that there is no great difference in the prevalence for back pain at the time of the survey or rather within the 12 months before. Besides, the data for back pain from the AT-HIS 2006–07 did not seem conspicuous and were similar and corresponded roughly to the data of the Microcensus surveys. Hence, the effect of different definitions is probably minimal and only accounts for a small change in the back pain prevalence during the study period. For measuring obesity, the participants were asked to indicate their body height (without wearing shoes) in centimetres and their body weight (without wearing clothes) in kilograms. The BMI for each subject was calculated by dividing body weight in kilograms by the square of body height in meters (kg/m^2^). According to the WHO [20], obesity was defined as a BMI greater than 30 kg/m^2^. To stratify the outcomes by age, adult subjects were categorized into the following four age groups: 20–34 years, 35–54 years, 55–74 years and 75 years and older.

### Correcting for self-report bias

Self-reported data on weight and height may lead to a misclassification of BMI values and may induce bias in measuring obesity [23]. Therefore, based on the results of a preliminary validation study among Austrian residents [24], BMI correction factors were applied only to subjects 45 years and older given that variations between self-reported and measured BMI significantly increased only in older women and men. Correction factors for women: 45 to 59 years old: +0.41 kg/m^2^, 60 years and older: +1.09 kg/m^2^. Correction factors for men: 45 to 59 years old: +0.50 kg/m^2^, 60 years and older: +0.54 kg/m^2^.

Self-reported information about the presence of disorders is more valid when it comes to chronic illnesses. Health surveys based on self-reported data have thus been considered as a good instrument for measuring the prevalence of diseases or disorders [25]. Therefore, no correction for self-reported presence of back pain was made in this study.

### Data analysis

**Figure 1:**
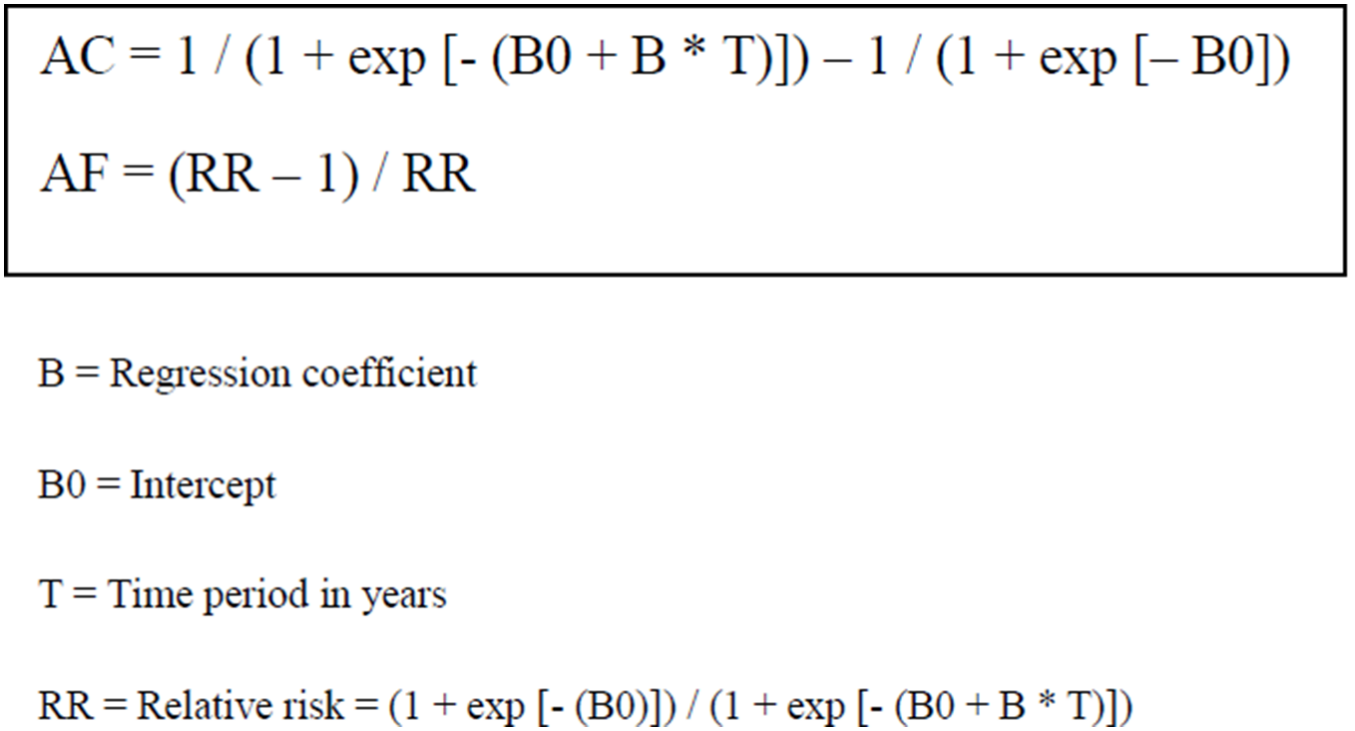
Formulas for computing the absolute change (AC) and the aetiologic fraction (AF).

Selected variables from all five surveys were fed into a common database. Crude prevalence and age-standardized prevalence were calculated, using the new European standard population for direct standardization in accordance with WHO guidelines [26]. Prevalence calculations were stratified by sex, age, and obesity. Chi-square tests were carried out for the whole study population and for subgroups, thereby analysing the statistical significance for the survey period. Figures representing the course of the prevalence of back pain, stratified by sex and obesity between 1973 and 2006–07, were created using Microsoft Office Excel 2007. To quantify trends in the prevalence of back pain, the percentages of absolute change (AC) were assessed. The aetiologic fraction (AF), a ratio measure, was computed to represent the subgroup with the greatest relative obesity risk. The AF denoted the percentage portion of the disease risk. To calculate the AC and AF, the prevalences of the first and last year (Pf and Pl, respectively) estimated by binary logistic regression models, were used. Binary logistic regression analyses were calculated for the whole study period with the dichotomous variable of back pain as dependent variable and the survey period as predictor. The correction variable for regression was age (in intervals of 5 years). The AC was defined as AC = Pl−Pf, and the AF was defined as AF = (Pl−Pf)/Pl. The precise formulas are presented in Figure 1. Statistical tests were two-sided and a p<0.05 was considered statistically significant. All statistical analyses were conducted using IBM SPSS Statistics for Windows version 21.0 (IBM Corp., Armonk, New York) and Stata/SE for Windows version 11.2 (StataCorp., College Station, TX, USA).

## Results

Table 1 shows that in 2006–07 the age-standardized prevalence of back pain was 32.9% among the general adult population in Austria, with the highest prevalence among obese subjects (36.2%). Overall, the prevalence was slightly higher in women than in men. Considering female and male adults in different age groups, the oldest group (≥75 years) suffered the most from back pain. Nearly half of the women in that age category reported back pain in the last survey. Among men the prevalence of back pain in 2006–07 was highest for those aged 55 to 74 years. When stratifying the outcomes of the most recent survey by age and obesity, we observed the highest prevalence of back pain in obese female (51.8%) and obese male (48.6%) adults aged 55 to 74 years. Among the non-obese the prevalence was highest for women aged 75 years and older (48.7%), and for men aged 55 to 74 years (43.4%) (Table 1).

**Table 1:** The prevalence of back pain in five health surveys in Austria stratified by sex, obesity and age.

				1973 (n = 55,814)			1983 (n = 38,835)			1991 (n = 35,093)			1999 (n = 34,731)			2006–07 (n = 14,318)		
Sex (n)	P value^a^			%	%	%	%	%	%	%	%	%	%	%	%	%	%	%
Age group (n)	Total	Obese	Non-obese	Total	Obese	Non-obese	Total	Obese	Non-obese	Total	Obese	Non-obese	Total	Obese	Non-obese	Total	Obese	Non-obese
**Total**(178,818)	p<0.001	p<0.001	p<0.001	14.8	19.1	14.2	16.7	21.2	16.2	19.2	24.4	18.7	25.0	33.7	23.7	34.3	42.8	32.8
**Total age-** **standardized**	p<0.001	p<0.001	p<0.001	13.9	16.3	13.6	16.5	18.5	16.4	19.0	22.0	19.0	24.3	28.7	23.5	32.9	36.2	32.2
20–34(50,509)	p<0.001	p<0.001	p<0.001	7.0	10.9	6.9	7.6	11.2	7.5	9.8	18.3	9.5	14.6	19.6	14.3	18.6	23.9	18.2
35–54(64,666)	p<0.001	p<0.001	p<0.001	15.5	17.3	15.2	19.3	22.4	19.0	22.3	23.5	22.2	26.0	30.7	25.4	33.6	38.0	32.9
55–74(49,112)	p<0.001	p<0.001	p<0.001	19.3	22.0	18.7	22.1	21.1	22.3	25.6	25.2	25.7	33.0	38.8	31.4	45.8	50.4	44.4
≥75(14,531)	p<0.001	p<0.001	p<0.001	19.8	22.0	19.5	21.7	27.9	21.0	23.9	31.9	23.0	29.6	39.1	28.1	46.4	48.4	46.0
**Women** **total** (96,017)	p<0.001	p<0.001	p<0.001	15.7	21.3	14.9	17.0	22.7	16.4	19.0	25.7	18.2	24.6	35.2	23.0	36.3	44.5	34.7
**Women** **total age-** **standardized**	p<0.001	p<0.001	p<0.001	14.2	18.3	13.8	16.4	20.0	16.1	18.5	22.0	18.2	23.6	29.0	22.6	34.3	35.7	33.7
20–34(25,140)	p<0.001	0.444	p<0.001	7.1	14.2	6.9	7.3	11.8	7.2	9.4	15.5	9.2	13.6	18.0	13.6	19.7	17.4	19.9
35–54(33,168)	p<0.001	p<0.001	p<0.001	15.3	18.8	14.3	18.8	23.8	18.2	20.7	25.0	20.3	24.7	30.9	23.9	34.9	39.4	34.3
55–74(27,967)	p<0.001	p<0.001	p<0.001	20.4	23.3	19.7	22.5	22.2	22.6	25.4	25.6	25.3	32.6	40.1	30.2	47.0	51.8	45.2
≥75(9,742)	p<0.001	p<0.001	p<0.001	22.1	25.0	21.7	22.7	28.9	21.9	25.4	34.5	24.2	30.5	39.9	28.7	49.3	51.2	48.7
**Men total**(82,801)	p<0.001	p<0.001	p<0.001	13.7	15.5	13.5	16.3	18.9	16.0	19.5	22.6	19.2	25.5	31.8	24.6	32.2	40.9	30.7
**Men total** **age-standardized**	p<0.001	p<0.001	p<0.001	13.2	13.5	13.2	16.5	16.3	16.6	19.7	21.0	19.8	24.9	28.0	24.5	31.2	35.6	30.5
20–34(25,368)	p<0.001	p<0.001	p<0.001	6.9	8.7	6.8	8.0	10.7	7.9	10.2	20.3	9.8	15.3	21.1	15.0	17.5	30.4	16.5
35–54(31,498)	p<0.001	p<0.001	p<0.001	15.6	15.5	15.6	19.8	21.0	19.7	24.0	22.1	24.2	27.4	30.5	26.9	32.3	36.8	31.5
55–74(21,146)	p<0.001	p<0.001	p<0.001	17.7	18.9	17.5	21.6	18.9	22.0	25.9	24.2	26.1	33.4	36.7	32.7	44.6	48.6	43.4
≥75(4,789)	p<0.001	p<0.001	p<0.001	15.1	11.8	15.3	19.5	23.1	19.4	20.8	23.1	20.7	27.7	36.2	26.7	41.0	38.0	41.2

a according to the Chi-square test of period effect.

**Figure 2:**
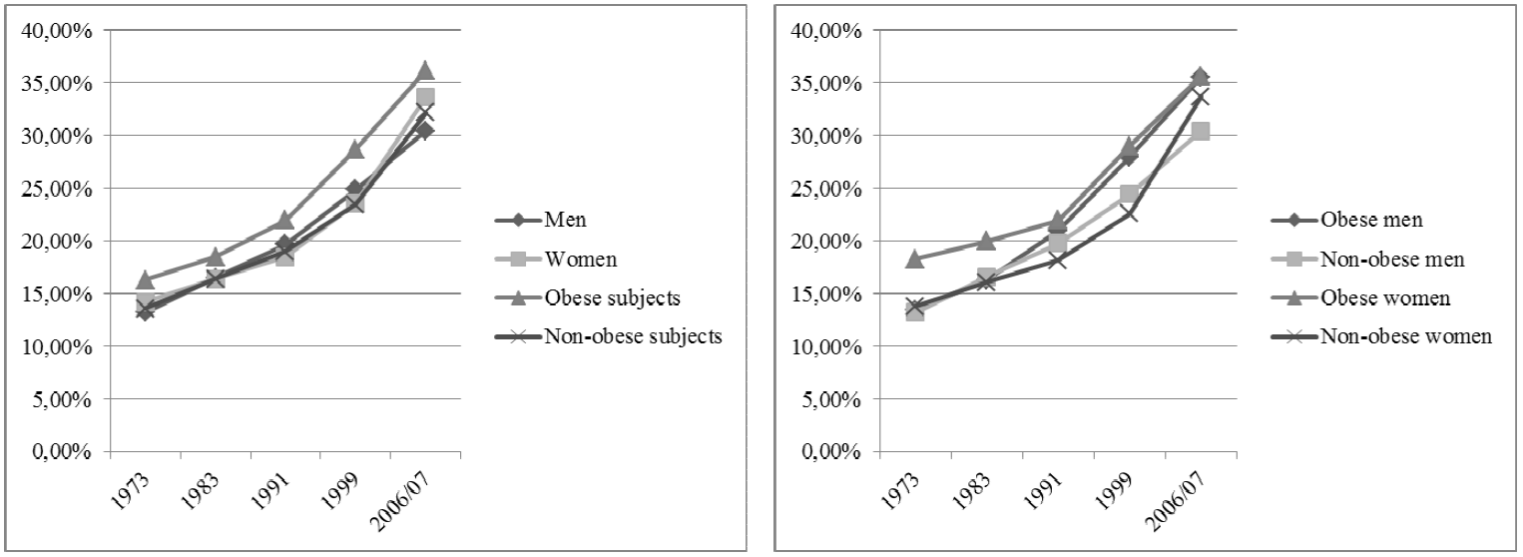
The age-standardized prevalence of back pain in Austria by sex and obesity in five health surveys.

In the period 1973 to 2006–07 the prevalence of back pain increased steadily across all subgroups (Table 1). Figure 2 illustrates how the age-standardized prevalence of back pain rose from survey to survey, among women and men and among obese and non-obese subjects. Overall, obese subjects were still the most affected by back pain during the study period. The continuous increase in the prevalence of back pain among obese and non-obese women and men is also illustrated in Figure 2. The outcomes for obese women demonstrated the highest prevalence across all surveys with an approximation in the prevalence of back pain among obese men in 2006–07. In the period 1999 to 2006–07 the prevalence of back pain strongly increased among women not suffering from obesity.

For the whole study population the increase in the prevalence of back pain represented by the absolute change (AC) was 19.4%. Obese subjects showed the highest growth between 1973 and 2006–07. Overall, the strongest AC was calculated for obese men (25.6%) and the lowest for non-obese men (16%). The greatest risk for back pain presented by the aetiologic fraction (AF) was found for obese men (61.8%). The lowest AF was observed among obese women (50%) (Table 2).

**Table 2:** Absolute changes (AC) and aetiologic fractions (AF) of the prevalence of back pain for the period 1973 to 2006–07 in Austria by sex and obesity (adjusted for age).

Predictor	AC back pain in %	AF back pain in %	P value*
**Total**	19.4	54.6	<0.001
** Obese**	25.0	53.7	<0.001
** Non-obese**	18.4	53.9	<0.001
**Women**	19.7	52.6	<0.001
** Obese**	23.3	50.0	<0.001
** Non-obese**	19.5	52.4	<0.001
**Men**	17.3	58.0	<0.001
** Obese**	25.6	61.8	<0.001
** Non-obese**	16.0	57.1	<0.001

*p value for period effect (from logistic regression analyses).

## Discussion

According to the age-standardized prevalence, about one third of the Austrian adult population suffered from back pain in 2006–07. Similar rates were found in a comparable study. A Greek population study estimated a self-reported 1-month prevalence to be 31.7% [7]. In Belgium the point prevalence of back pain was estimated to be 33% among adults. However, this study dates back to the early 1990ies [27]. Compared to other European countries the prevalence of back pain was quite high in this study [2], [5], [10], [28]. Hoy et al. [2], in their meta-analysis of summarized evidence from 165 studies in 54 countries, stated a pooled estimate of the mean point prevalence of low back pain to be 11.9%.

Considering the overall trend, the prevalence of back pain in Austria was not always so high. A progression of back pain was observed and thus the prevalence rose with every survey. Data from cross-sectional surveys conducted over 40 years among English adults were investigated by Harkness et al. [28] They found a strong increase in the point prevalence of self-reported back pain. While the prevalence of low back pain was 9.1% for both women and men in 1956, it went up to 18.2% for women and 17.8% for men in 1995. Slightly higher rates than in the 1990ies were observed in our study. However, no other current studies were found that examined long-term trends in the prevalence of back pain. Therefore, comparisons for long-term back pain trends are not possible.

We believe that there is a series of factors that may have led to this strong increase in the prevalence of back pain in Austria. One reason is that the demographic has changed during the study period in Austria. There was a change in the age structure of the population in favor of the older age groups and the demographic aging has led to a more frequent occur of chronic diseases, including back pain. Over the period, the work situation has also changed. Increased workload and increased sedentary activities may have contributed to a higher prevalence of back pain [29]. The increase in back pain is probably attributed partly to the rise of BMI and obesity prevalence. Among obese subjects an almost linear trend in the increasing prevalence is discernible from 1991 onwards [21]. Another reason could be that there was an increase of subjects with mental disorders, which causes future episodes of pain [30]. An assumption is also that the willingness to report pain symptoms increased. This may be due to cultural factors as changes in the attitudes to report their disorders [29], which is also noticeable by increased sickness reporting [13]. Furthermore an increased awareness of pain symptoms by patients and health professionals could have been contributed to the rise in the prevalence of back pain. It is unclear if the high prevalence in the most recent surveys indicates a true increase or represents a rise in unclear diagnosis [29]. Overall, the evidence suggests that the real increase in back pain is likely to be somewhat lower than the self-reported figures.

The analysis of subgroups in this study showed interesting results. The literature indicates back pain is more common among female and older persons [5], [2], [7], [17], [27], [31]. In line with earlier studies, women showed a higher prevalence of back pain than men [32], [33]. Results from a systematic review also showed that back pain worldwide more often concerns women than men [2]. Possible underlying reasons for the gender difference were investigated in Germany [31]. When investigating different factors related to back pain (e.g. BMI, age) it was not possible to reduce or explain the gender difference. They recommended exploring rarely investigated constructs, such as anxiety and considering anatomic differences in muscle strength. While the prevalence of back pain was highest among women in Austria, the strongest increase and the greatest risk were found for men. In Bulgaria the risk of getting back disorders was also higher in men [34]. This could be due to the fact that women paid more attention to a healthy lifestyle, including back exercises, in the last decades as social norms generally make women more cautious about their body.

Considering different age groups the prevalence for back pain increases with age, with the highest incidence in the third decade [11], [28]. In the oldest age group the prevalence decreases [1], [35], which was also true for this study. There are a whole range of factors behind this phenomenon: decreased pain perception or increased pain tolerance, existence of other health problems with higher priority, increasing influence of mental health problems and the exclusion from studies of older individuals living in nursing homes [1]. It is striking that in young obese men the prevalence is already very high in contrast to young obese women. The latest survey showed one third of all 20–34 year old obese men to suffer from low back pain, while the same applies to only half of the non-obese peers. Lean body mass is found more often in younger than in older subjects. Therefore, the lower back pain prevalence among young men with a high BMI may be due to increased muscle mass [36]. However, it should be noted that the women are clearly catching up at a later age, which is why the overall prevalence of low back pain is generally somewhat higher among women.

Studies reported that obesity is associated with a higher prevalence of low back pain [10], [15], [34], [37], which is more pronounced in women [18], [32], [38]. Study results showing higher prevalence of back pain for obese than for non-obese subjects are in accordance with our findings. We observed the highest prevalence among obese women, however the increase of and the greatest risk for back pain were highest among obese men. Therefore, special emphasis should be placed on obese individuals when planning low back pain prevention strategies. A moderate level of physical activity is recommended to prevent back pain [17], [18].

### Strengths and Limitations

One limitation was that only self-reported data were available for Austria. This disadvantage was compensated by correcting the self-reported BMI [24]. Another limitation was due to the fact that the last survey used a different definition for identifying back pain. This restriction is described in more detail in the Method section. Furthermore, the literature showed that low back pain is more often associated with obesity [17], [18]. In our study only data concerning general back pain was available for analysis. In addition the data concerning back pain did not include any information on the severity and frequency of the pain. More precise data would be needed in order to develop an appropriate therapeutic approach.

When interpreting the prevalence of diseases, it is recommended to standardize crude rates since populations may differ in their age composition. However, consideration should be given to the fact that age-adjusted rates are partially derived from a reference population, so they do not precisely describe the study population. It is a strength that crude and age-standardized prevalence was mentioned in this study. Another strength comprises the unique database with a large number of subjects included, enabling us to obtain statistically reliable data in subgroups. Examination over such a long investigation period allowed an accurate assessment of the development of this public health problem, which does represent a major advantage.

### Conclusion

In conclusion, back pain strongly increased in the last decades and currently represents a widespread public health problem in Austria. The outcomes further indicate that there was an increase in the prevalence of back pain among all investigated subgroups, with the highest prevalence among obese women. However, obese men showed the highest increase of and the greatest risk for back pain during the study period. Our findings confirm the effect of a high BMI as a risk factor for back pain in the general adult population. The link between obesity and back pain underlies the importance of promoting preventive measures to reduce the incidence of obesity. We recommend that this worrying trend be monitored throughout the Austrian population and that preventive measures be implemented for specific target groups, as obese subjects. The results could be used to plan controlled promotion programs among adults suffering from back pain.

It should be noted that this study did not identify the nature of the stated relationships. The precise process leading to the relationship between low back pain and investigated variables should be clarified through further studies in order to combine our epidemiologic results with the processes leading to the genesis of back pain. Prospective studies are needed to confirm our results and investigate causal relationships.
